# Health-related Quality of Life in Women with Cervical Cancer

**DOI:** 10.1055/s-0039-1683355

**Published:** 2019-03-07

**Authors:** Larissa Nascimento dos Santos, Luciana Castaneda, Suzana Sales de Aguiar, Luiz Claudio Santos Thuler, Rosalina Jorge Koifman, Anke Bergmann

**Affiliations:** 1 Instituto Federal do Rio de Janeiro, Rio de Janeiro, RJ, Brazil; 2 Instituto Nacional de Câncer, Rio de Janeiro, RJ, Brazil; 3 Fundação Oswaldo Cruz, Rio de Janeiro, RJ, Brazil

**Keywords:** uterine cervical neoplasms, quality of life, cross-sectional studies, data collection, câncer do colo do útero, qualidade de vida, estudos transversais, coleta de dados

## Abstract

**Objective** To analyze the factors associated with health-related quality of life (HRQoL) in women with cervical cancer (CC) in a single center in Rio de Janeiro, state of Rio de Janeiro, Brazil.

**Methods** A cross-sectional study in women with a diagnosis of CC followed-up in the gynecology outpatient clinic of the Hospital do Câncer II (HCII, in the Portuguese acronym) of the Instituto Nacional de Câncer (INCA, in the Portuguese acronym). The data were collected from March to August 2015. Women with palliative care, communication/cognition difficulty, undergoing simultaneous treatment for other types of cancer, or undergoing chemotherapy and/or radiation therapy were excluded. For the evaluation of the HRQoL, a specific questionnaire for women with CC was used (Functional Assessment of Cancer Therapy – Cervix Cancer [FACT-Cx]). The total score of the questionnaire ranges from 0 to 168, with higher scores indicating a better HRQoL.

**Results** A total of 115 women were included in the present study, with a mean age of 52.64 years old (standard deviation [SD] = 12.13). The domains of emotional (16.61; SD = 4.55) and functional well-being (17.63; SD = 6.15) were those which presented the worst scores. The factors that had an association with better HRQoL in women with CC were having a current occupation, a longer time since the treatment and diagnosis, and women who had undergone hysterectomy.

**Conclusion** Considering the domains of HRQoL of the women treated for cervical cancer, a better score was observed in the domains of physical and social/family well-being. For most domains, better scores were found between those with a current occupation, with a longer time after the diagnosis and treatment, and among those who had undergone a hysterectomy.

## Abstract (pt)

**Resumo:**

**Objetivo** Analisar os fatores associados à qualidade de vida em mulheres com câncer de colo do útero tratadas em um hospital de referência no Rio de Janeiro, RJ, Brasil.

**Métodos** Estudo transversal em mulheres com diagnóstico de câncer do colo do útero em seguimento ambulatorial no Hospital de Câncer II (HCII) do Instituto Nacional de Câncer (INCA). Os dados foram coletados no período de março a agosto de 2015. Foram excluídas as mulheres em cuidados paliativos, com dificuldade de comunicação/cognição, que estavam em tratamento simultâneo para outros tipos de câncer, ou em quimioterapia e/ou radioterapia. Para a avaliação da qualidade de vida relacionada à saúde, foi utilizado um questionário específico para mulheres com câncer de colo do útero (Avaliação Funcional da Terapia do Câncer – Câncer Cervical (FACT-Cx, na sigla em inglês). O escore total do questionário varia de 0 a 168, com escores mais altos indicando melhor qualidade de vida relacionada à saúde.

**Resultados** Foram incluídas 115 mulheres com uma média de idade de 52,64 anos (desvio padrão [DP] = 12,13). Os domínios de questões emocionais (16,61; DP = 4,55) e de bem-estar funcional (17,63; DP = 6,15) foram os que apresentaram os piores escores. Os fatores que tiveram associação com melhor qualidade de vida relacionada à saúde em mulheres com câncer de colo do útero foram ocupação atual, maior tempo após o tratamento e diagnóstico, e mulheres que haviam sido submetidas a histerectomia.

**Conclusão** Considerando os domínios da qualidade de vida relacionada à saúde de mulheres tratadas para câncer do colo do útero, foi observado melhor escore nos domínios de bem-estar físico e social. Para a maioria dos domínios, os melhores escores foram observados entre aquelas com ocupação atual, com mais tempo após o diagnóstico e tratamento, além daquelas que se submeteram a histerectomia.

## Introduction

Cervical cancer (CC) is a significant public health problem. In Brazil, it is the 4^th^ most common cancer among women,^1^ and in the world, the 3^rd^, with an incidence of > 520,000 cases. In 2012, CC was responsible for > 260,000 deaths worldwide. Estimates indicate that, of these, ∼ 230,000 occurred in less developed countries.^2^ In middle- and low-income countries, the diagnoses of CC are performed too late. Women ≥ 50 years old, living without a partner, and with a low educational level, had a higher risk of receiving the diagnosis of advanced stage CC.^3^ In 37,638 Brazilian women with CC, the determination in late clinical stages was observed in 70.6% of the cases, with squamous cell carcinoma being the most common type associated with ages ≥50 years old, black skin color, and low educational level.^4^ The impact of the treatment of CC can lead to losses in the quality of life (QoL) of the patients. The presence of side effects such as fatigue, diarrhea, nausea, urinary incontinence, lymphoedema, vaginal stenosis, lack of vaginal lubrication, dyspareunia, sensory problems, sleep disorders, stress, and depression is common.^5^ ^6^ ^7^ In addition to the impact of the diagnosis of cancer, the consequence of the complications and of the changes in reproductive and hormonal functions affect the identity of the woman.^8^ ^9^ ^10^

Health-related quality of life (HRQoL) is defined by the World Health Organization (WHO) as “the perception of the individual about his position in life, in the context of culture and value systems in which he lives, and about his goals, expectations, standards, and concerns.”^11^ The measurement of HRQoL in women with CC becomes critical. Aspects that not only restrict the clinical treatment but also encompass information on the social participation, on the mental state, and on the functionality of the woman should be included in assessment protocols.^12^

In this context, the present study aims to analyze the factors associated with HRQoL in women with CC in a single center in Rio de Janeiro, state of Rio de Janeiro, Brazil.

## Methods

A cross-sectional study was conducted. Women diagnosed with CC at the Hospital do Câncer II (HCII, in the Portuguese acronym) of the Instituto Nacional de Câncer (INCA, in the Portuguese acronym) from March to August 2015 were included. Patients who attended the gynecology outpatient clinic for follow-up consultation were recruited. Women with palliative care, with communication/cognition difficulty, who were undergoing simultaneous treatment for other types of cancer, or who were undergoing chemotherapy and/or radiation therapy were excluded.

The eligible individuals had the objectives of the present study explained to them and signed the informed consent. The present study was approved by the Ethics and Research Committee of the INCA (CAAE 36438414.6.0000.5274).

The sociodemographic and clinical variables were obtained through the interview and by consulting the medical records of the patients. The instruments for the collection of data were a questionnaire including sociodemographic and clinical variables, and a specific questionnaire of HRQoL for women with CC (Functional Assessment of Cancer Therapy – Cervix Cancer [FACT-Cx]). This instrument was validated for the Brazilian population.^13^ The time interval (years) between the first treatment for CC and the measurement of the HRQoL was considered as “time since treatment”, and the time interval (years) between the diagnosis of CC and the measurement of the HRQoL was considered as “time since diagnosis.”

The FACT-Cx assesses the functioning and satisfaction of women with CC, regarding the previous 7 days. It consists of 42 items comprising the FACT-General (FACT-G) questionnaire (27 questions) and the CC-specific subscale (15 items). The 15 specific subscale items about CC refer to the “additional concerns” domain. The questionnaire is scored on a Likert-type scale of 0 (not at all) to 4 (very much). Some items had built-in negative phrases and, in these cases, the scores were reversed. The total scores range from 0 to 168, with higher scores indicating a better HRQoL.

The descriptive analysis of the population under study was performed using the mean (±standard deviation [SD]) and median (minimum–maximum) for continuous variables, and frequency distribution for categorical variables. To evaluate the outcome (score from the domains of QoL), a simple linear regression was performed, and the variables that presented *p* < 0.20 were selected for the multiple linear regression model. The value of the β coefficient is the difference in means. The adjusted model was composed of the variables that showed statistical significance (*p* < 0.05). All of the analyses were performed using IBM SPSS Statistics for Windows, Version 23.0 (IBM Corp., Armonk, NY, USA).

## Results

In the present study, a total of 115 women were included. The mean age of the patients was 52.64 years old (±12.13), with a mean body mass index (BMI) of 27.18 (±5.97). At the time of the interview, 40.0% of the patients had incomplete elementary school education and had a mean of 3 children (±2.21). The women were married or in a stable relationship (47.0%), did not have an occupation (64.3%), and reported not smoking (90.4%) or consuming alcohol (82.6%) currently. The patients were classified as up to stage IB (41.7%), and 63.5% had undergone radiotherapy and chemotherapy (Table 1).

**Table 1:** Sociodemographic and clinical characteristics of the study population (*n* = 115)

Variables	*n*	%
Schooling/ educational level		
Incomplete elementary school education	72	62.5
Complete elementary school education	43	37.5
Marital status		
Married/stable relationship	54	47.0
Divorced	19	16.5
Widowed	21	18.3
Single	21	18.3
Race		
White	43	37.4
Other	72	62.6
Occupation		
Yes	41	35.7
No	74	64.3
Currently smoking		
Yes	11	9.6
No	104	90.4
Alcohol consumption (previous 7 days)		
Yes	20	17.4
No	95	82.6
Stage (FIGO)		
IA	12	10.4
IB	36	31.3
IIA	17	14.8
IIB	19	16.5
IIIA	13	11.3
IIIB	18	15.7
Treatment		
Chemotherapy + radiotherapy	73	63.5
Hysterectomy	42	36.5

Abbreviations: FIGO, International Federation of Gynecology and Obstetrics.

In Table 2, the HRQoL (FACT-Cx) scores are presented. The lowest means of the domains were observed in the emotional well-being (16.61 ± 4.55), and in the functional well-being (17.63 ± 6.15), and the highest factors were in the physical well-being (19.26 ± 5.63), and in the social/family well-being (18.20 ± 5.78). The additional concerns domain presented a mean score of 41.69 (±8.49), and the total score of the FACT-Cx was 112.15 (±22.91).

**Table 2:** Health-related quality of life scores in women with cervical cancer (*n* = 115)

FACT-Cx	Mean (SD)	Median	Minimum	Maximum	Score range
Physical well-being (PWB)	19.26 (5.63)	19.0	4.0	28.0	0–28
Social/Family well-being (SWB)	18.20 (5.78)	18.0	2.0	28.0	0–28
Emotional well-being (EWB)	16.61 (4.55)	16.0	6.0	24.0	0–24
Functional well-being (FWB)	17.63 (6.15)	18.0	2.0	28.0	0–28
Additional Concerns	41.69 (8.49)	42.0	20.0	60.0	0–60
Total FACT-Cx	112.15 (22.91)	109.5	61.0	168.0	0–168

Abbreviations: FACT-Cx, Functional Assessment of Cancer Therapy – Cervix Cancer; SD, Standard Deviation.

The variables associated with each FACT-Cx domain are presented in Table 3. In each area of QoL, the variables that presented *p* < 0.20 were selected for the multiple linear regression model. The independent factors associated with HRQoL in every domain are shown in Table 4. In the physical well-being domain, women with an occupation had a HRQoL score 2.39 points higher than women without a profession. In the social/family well-being domain, better HRQoL scores were reported in women with > 2 years since the diagnosis of CC (3.53 points), no children (4.88 points), and who had been treated with hysterectomy (2.57 points). Better scores in the domains of emotional and functional well-being were observed for women with > 2 years since the diagnosis or since the treatment (1.96 and 4.25, respectively). In the domain of additional concerns specific to CC, women with an occupation had higher HRQoL scores (3.54 points). In the total score for the FACT-Cx, having a job (14.01 points), and having undergone hysterectomy (8.82 points) were both associated with a better HRQoL.

**Table 3:** Association of health-related quality of life, sociodemographic and clinical variables (univariate analysis)

Variables	Physical well-being (PWB)		Social well-being (SWB)		Emotional well-being (EWB)		Functional well-being (FWB)		Additional Concerns		Total FACT-Cx	
	Mean (SD)	*p-value*	Mean (SD)	*p-value*	Mean (SD)	*p-value*	Mean (SD)	*p-value*	Mean (SD)	*p-value*	Mean (SD)	*p-value*
**Age (years old)**												
< 50	18.96 (5.16)	0.633	16.93 (6.65)	0.500	16.20 (4.40)	0.432	16.34 (5.96)	0.600	40.61 (7.81)	0.600	109.84 (22.78)	0.248
≥50	19.47 (5.97)		19.13 (4.90)		16.88 (4.66)		18.53 (6.17)		42.55 (8.97)		113.96 (23.06)	
**Stage (FIGO)**												
< IIB	18.74 (5.39)	0.387	17.60 (5.82)	0.341	15.86 (4.65)	**0.123**	16.90 (6.43)	0.263	40.67 (7.96)	0.256	109.84 (22.78)	**0.133**
≥ IIB	19.66 (5.83)		18.67 (5.75)		17.19 (4.42)		18.20 (5.91)		42.57 (8.90)		113.96 (23.06)	
**Treatment**												
CT + RT	18.48 (5.35)	0.500	17.21 (6.13)	**0.020**	16.36 (4.78)	0.456	17.16 (6.49)	0.282	40.52 (8.22)	**0.058**	108.21 (20.99)	**0.024**
Hysterectomy	20.62 (5.93)		19.85 (4.77)		17.02 (4.16)		18.45 (5.50)		43.81 (8.68)		118.86 (24.73)	
**Children**												
No	21.44 (6.00)	0.228	23.37 (4.41)	**0.008**	18.22 (4.55)	0.237	21.00 (6.59)	0.880	44.13 (10.41)	0.402	128.43 (23.06)	**0.051**
Yes	19.08 (5.60)		17.79 (5.69)		16.46 (4.54)		17.35 (6.06)		41.49 (8.34)		110.92 (22.55)	
**Time since treatment (years)**												
≤ 2	18.80 (4.89)	0.325	16.97 (5.88)	**0.008**	16.00 (4.50)	**0.115**	15.75 (5.66)	**<0.001**	41.25 (7.61)	0.538	108.51 (20. 81)	**0.056**
> 2	19.84 (6.54)		19.89 (5.25)		17.35 (4.55)		20.00 (5.97)		42.30 (9.62)		117.39 (24.97)	
**Time since diagnosis (years)**												
≤ 2	18.83 (4.12)	0.451	16.29 (5.88)	**0.001**	15.54 (4.54)	**0.021**	15.45 (5.13)	**<0.001**	41.72 (7.02)	0.975	108.27 (19.91)	**0.097**
> 2	19.63 (6.68)		19.88 (5.18)		17.50 (4.40)		19.50 (6.38)		41.67 (9.72)		115.88 (25.09)	
**Education (elementary school)**												
Incomplete	19.11 (5.89)	0.814	17.14 (5.80)	**0.122**	16.20 (4.68)	0.432	17.91 (5.67)	0.694	42.63 (7.99)	0.364	110.38 (22.14)	0.541
Complete	19.36 (5.50)		18.89 (4.48)		16.88 (4.48)		17.45 (6.49)		41.08 (8.81)		113.28 (23.50)	
**Variables**	**Physical well-being (PWB)**		**Social well-being (SWB)**		**Emotional well-being** **(EWB)**		**Functional well-being** **(FWB)**		**Additional Concerns**		**Total FACT-Cx**	
	**Mean (SD)**	***p-value***	**Mean (SD)**	***p-value***	**Mean (SD)**	***p-value***	**Mean (SD)**	***p-value***	**Mean (SD)**	***p-value***	**Mean (SD)**	***p-value***
**With partner**												
Yes	19.57 (5.88)	0.577	17.24 (5.90)	**0.085**	16.70 (4.33)	0.840	17.43 (6.13)	0.734	42.55 (6.92)	0.315	112.76 (21.06)	0.792
No	18.98 (5.45)		19.15 (5.55)		16.52 (4.78)		17.82 (6.22)		40.87 (9.76)		111.54 (24.82)	
**Race**												
White	18.67 (6.17)	0.391	18.15 (5.53)	0.944	15.79 (4.84)	**0.138**	17.28 (5.67)	0.634	40.87 (8.78)	0.448	110.03 (24.16)	0.48
Other	19.61 (5.31)		18.23 (5.96)		17.10 (4.33)		17.85 (6.45)		42.18 (8.34)		113.40 (22.25)	
**Occupation**												
No	18.41 (5.48)	**0.028**	17.27 (5.08)	**0.024**	15.99 (4.12)	**0.048**	16.70 (5.96)	**0.028**	40.43 (7.46)	**0.041**	106.83 (19.49)	**0.001**
Yes	20.80 (5.66)		19.87 (6.61)		17.75 (5.12)		19.32 (6.22)		43.97 (9.80)		122.03 (25.67)	
**BMI**												
Adequate	18.21 (5.04)	**0.123**	17.08 (5.18)	**0.122**	16.40 (4.04)	0.721	15.88 (5.39)	**0.018**	41.16 (8.40)	0.629	107.37 (19.89)	**0.127**
Overweight and obese	19.89 (5.91)		18.86 (6.04)		16.72 (4.85)		18.68 (6.37)		42.00 (8.59)		114.72 (24.14)	

Abbreviations: BMI, Body mass index; CT, chemotherapy; FACT-Cx, Functional Assessment of Cancer Therapy – Cervix Cancer; FIGO, International Federation of Gynecology and Obstetrics; RT, radiotherapy; SD, standard deviation.

The variables in bold were selected for preparation of the multiple linear regression model (*p* < 0.20).

In all of the functional scales, the higher the score, the better the quality of life.

**Table 4:** Factors associated with health-related quality of life in women with cervical cancer (*n* = 115)

Domains of quality of life	Difference in means (β coefficient)	95% CI	*p-value*
Physical well-being (PWB)			
Occupation (Yes)	2.39	0.26–4.54	0.028
Social well-being (SWB)			
Time since diagnosis (> 2 years)	3.53	1.53–5.53	0.001
Having Children (No)	4.88	1.05–8.721	0.013
Treatment (Hysterectomy)	2.57	0.51–4.63	0.015
Emotional well-being (EWB)			
Time since diagnosis (> 2 years)	1.96	0.30–3.63	0.021
Functional well-being (FWB)			
Time since treatment (> 2 years)	4.25	2.09–6.41	< 0.001
Additional Concerns			
Occupation (Yes)	3.54	0.14–6.94	0.041
Total FACT-Cx			
Occupation (Yes)	14.01	4.98–23.05	0.003
Treatment (Hysterectomy)	8.82	- 0.10–17.75	0.053

Abbreviations: CI, confidence interval; CT, chemotherapy; RT, radiotherapy.

## Discussion

For most of the domains of HRQoL, the best scores were observed in women with a longer time elapsed since the time of diagnosis and treatment, in addition to those who had undergone hysterectomy.

Our results show that ∼ 42% of the patients were diagnosed in the initial stages of CC (up to stage IB), which was in contrast to what has been observed in another study in Brazil, with a sample of 4,950 cases, in which 69.4% of the cases had locally advanced disease (stages II and III), and stage I represented only 27.9% of the cases.^14^ In another survey conducted in Brazil, 55% of the women were in stage IIIb.^15^ In contrast, a study performed in China with 400 women, 61% were diagnosed in the initial stage (I),^16^ while another survey in Korea with 860 women noted that 66.8% of the women were in stage I.^17^

Women who have undergone hysterectomy presented better QoL scores. We could speculate that women for whom hysterectomy was indicated had a better prognosis than women for whom radiotherapy was indicated. However, a recent Cochrane systematic review found insufficient evidence that hysterectomy with radiation, with or without chemotherapy, improves the survival of women with locally advanced CC who are treated only with radiotherapy or chemoradiotherapy.^16^

Around 63% of the participants in our study were undergoing radiotherapy and chemotherapy, as has also been observed in a Chinese study, in which 57.1% and 77.1% of the patients also received radiotherapy and chemotherapy, respectively.^17^ In the survey by Osann et al,^18^ it was observed that patients who received radiation, with or without chemotherapy, had a worse HRQoL in comparison with patients who just underwent surgery. In a systematic review, radiation therapy was also associated with a worse HRQoL.^19^

Our results demonstrate that the best HRQoL domains were physical and social/family well-being, as in the studies of Zhou et al^17^ and of Ding et al.^20^ The worst scores were noted for the emotional well-being domain, which can be related to psychosocial factors and depression, since it is observed that women feel more incapable after treatment, mainly concerning housework.^19^ ^21^

The patients with an occupation at the time of the interview presented better HRQoL scores in physical well-being, additional concerns, and in the total FACT-Cx score. This finding corroborates the results of an Italian study, which showed that women with locally advanced CC who were unemployed had the worst ratings for HRQoL in all domains.^22^ In a multicentre study in Korea, which included 858 women, all of the QoL scores were better for women with an occupation.^23^ Women with a time since diagnosis > 2 years showed better HRQoL scores in the domains of social/family and of emotional well-being, and those with a time since treatment of > 2 years showed better HRQoL scores in the functional well-being domain. A study conducted in Taiwan^24^ showed that the older survivors and those with a longer time since treatment had worse global HRQoL scores; however, this was a study with a mean age that was higher than ours, and the results may be related to senescence rather than just to the time since treatment. In the study by Mantegna et al,^25^ it was reported that the longer the time since both diagnosis and treatment, the better the HRQoL, which is in line with our results.

This is a cross-sectional study with no internal comparison group. As possible limitations, we emphasize that, because it is a cross-sectional study, it is not possible to evaluate the temporality between the exposure variables (occupation and treatment) and HRQoL. Because it is a study conducted in a single center specialized in oncological surgery, the chance of systematic bias is lower, which provides a better internal validity. However, bias selection as to nonregistration of refusals and to total invitations can be present. Therefore, there is a decrease in external validity, and these results should be generalized to populations with similar characteristics (i.e., low socioeconomic level, users of the public health system [SUS, in the Portuguese acronym]).

The low socioeconomic level of our sample and the persistent inequities of health system in Brazil may also be associated with negative influences in HRQoL. Cancer is no longer a disease with a primary outcome of mortality. The sequelae of the disease may be caused by the disease itself, by its treatment, or even by a combination of the two. In women with CC, the diagnosis and treatment can result in a context of acute stress. Deterioration in QoL can occur in chronic stress situations, and this can have substantial effects on the well-being of these women.

## Conclusion

Considering the domains of HRQoL of women treated for CC, a better score was observed in the domains of physical and of social/family well-being. For most domains, better scores were found between those with a current occupation, with more time since diagnosis and treatment, and among those who had undergone hysterectomy.
